# A Correspondence Between Solution-State Dynamics of an Individual Protein and the Sequence and Conformational Diversity of its Family

**DOI:** 10.1371/journal.pcbi.1000393

**Published:** 2009-05-29

**Authors:** Gregory D. Friedland, Nils-Alexander Lakomek, Christian Griesinger, Jens Meiler, Tanja Kortemme

**Affiliations:** 1 Graduate Group in Biophysics, University of California San Francisco, San Francisco, California, United States of America; 2 Department of Bioengineering and Therapeutic Sciences, University of California San Francisco, San Francisco, California, United States of America; 3 California Institute for Quantitative Biosciences, University of California San Francisco, San Francisco, California, United States of America; 4 Department for NMR-based Structural Biology, Max-Planck Institute for Biophysical Chemistry, Goettingen, Germany; 5 Center for Structural Biology, Vanderbilt University, Nashville, Tennessee, United States of America; National Cancer Institute, United States of America and Tel Aviv University, Israel

## Abstract

Conformational ensembles are increasingly recognized as a useful representation to describe fundamental relationships between protein structure, dynamics and function. Here we present an ensemble of ubiquitin in solution that is created by sampling conformational space without experimental information using “Backrub” motions inspired by alternative conformations observed in sub-Angstrom resolution crystal structures. Backrub-generated structures are then selected to produce an ensemble that optimizes agreement with nuclear magnetic resonance (NMR) Residual Dipolar Couplings (RDCs). Using this ensemble, we probe two proposed relationships between properties of protein ensembles: (i) a link between native-state dynamics and the conformational heterogeneity observed in crystal structures, and (ii) a relation between dynamics of an individual protein and the conformational variability explored by its natural family. We show that the Backrub motional mechanism can simultaneously explore protein native-state dynamics measured by RDCs, encompass the conformational variability present in ubiquitin complex structures and facilitate sampling of conformational and sequence variability matching those occurring in the ubiquitin protein family. Our results thus support an overall relation between protein dynamics and conformational changes enabling sequence changes in evolution. More practically, the presented method can be applied to improve protein design predictions by accounting for intrinsic native-state dynamics.

## Author Summary

Knowledge of protein properties is essential for enhancing the understanding and engineering of biological functions. One key property of proteins is their flexibility—their intrinsic ability to adopt different conformations. This flexibility can be measured experimentally but the measurements are indirect and computational models are required to interpret them. Here we develop a new computational method for interpreting these measurements of flexibility and use it to create a model of flexibility of the protein ubiquitin. We apply our results to show relationships between the flexibility of one protein and the diversity of structures and amino acid sequences of the protein's evolutionary family. Thus, our results show that more accurate computational modeling of protein flexibility is useful for improving prediction of a broader range of amino acid sequences compatible with a given protein. Our method will be helpful for advancing methods to rationally engineer protein functions by enabling sampling of conformational and sequence diversity similar to that of a protein's evolutionary family.

## Introduction

It has long been known that a protein's native state is best represented as an ensemble of conformations rather than as a single structure [1]. Conformational ensembles provide a detailed structural picture of protein dynamics. As motions are crucial for many aspects of protein function, such as molecular recognition [2]–[4] and catalysis [5]–[10], an ensemble description of proteins is also useful for improving applications of molecular modeling such as protein-small molecule [11] and protein-protein docking methods [12],[13] as well as protein design [14]–[19].

Two related concepts characterizing and interpreting properties of protein conformational ensembles have been proposed: The first suggests a correspondence between the conformational heterogeneity present in crystal structures and the native-state dynamics of proteins observed in simulations and using nuclear magnetic resonance (NMR) measurements. Several studies provide support for this idea. Zoete et al. concluded that the conformational changes present in a large number of crystal structures of HIV-1 protease reflect the inherent flexibility of the protein [20]. Vendruscolo and coworkers showed [21] that side chain relaxation order parameters, reflecting motions on the picosecond to nanosecond time scale [22]–[28], could be described using ensembles of crystal structures of the same protein or proteins with high sequence identity. Similarly, modeling “Backrub” motions, a type of conformational change inspired by alternate side chain and backbone conformations observed in high-resolution crystal structures [29], has led to improvements in modeling NMR side chain relaxation order parameters [30], side chain conformations [31],[32] and structural changes upon mutation [31]. Lange et al. [4] showed that ensembles derived from ensemble-average-restraint molecular dynamics (MD) simulations of ubiquitin using Residual Dipolar Coupling (RDC) data describing picosecond to millisecond motions [33]–[41] encompassed conformations similar to those of ubiquitin in different crystal structures alone and in complex with different partner proteins. These findings support the idea that conformational states pre-existing in solution are selected upon binding. Strong experimental evidence for this conformational selection model was also provided earlier by Wright and colleagues [42] validating previous theoretical suggestions [43],[44].

The second concept proposes a link between the dynamics of a single protein and the conformational variability explored within its family of homologous proteins. This link was suggested based on the similar conformational variability observed in an MD simulation of myoglobin and in structures of different members of the globin family [45]. Similarly, Gaussian network models have suggested common dynamical features of proteins in the same family [46],[47]. Recently, Lee and colleagues proposed that side chain dynamics measured by NMR relaxation are conserved across members of the PDZ domain family [48]. Several studies extended the notion of a relationship between the dynamics of a single protein and properties of its homologs to the sequence level, showing that modeled sequences consistent with a single protein structure had characteristics in common with a multiple sequence alignment of the protein's natural family [49]. Further investigating the relation between protein dynamics and family sequence variability, other work suggested that sequence diversity [32] and overlap between modeled and evolutionarily observed sequences could be increased by incorporating conformational flexibility of the protein backbone [14]–[16],[50],[51].

To combine the two concepts outlined above, here we ask whether conformational ensembles reflecting variability observed in protein crystal structures of a single sequence can be simultaneously related to experimentally determined native-state solution dynamics of an individual protein, and to the conformational and sequence variability of the protein's family. To address these questions, we investigate two related hypotheses using ubiquitin as a model system: First, we test whether ensembles generated using the Backrub motional mechanism (“Backrub ensembles”), a model inspired by heterogeneity observed in experimental protein structures [29], capture properties of ubiquitin solution state dynamics derived from amide backbone RDC measurements in 23 alignment media [35]. The motions modeled using the Backrub mechanism are related to those described by the 1D-Gaussian Axial Fluctuation (GAF) approach, which has been used to model residual dipolar coupling (RDC) measurements [52]. Furthermore, we compare the structural variation in modeled Backrub ensembles to that seen in a set of 46 crystal structures of ubiquitin [4]. Second, we test whether the conformational heterogeneity present in Backrub ensembles that are consistent with the solution dynamics of an individual ubiquitin sequence resembles the structural diversity observed in the UBQ subfamily [53]. Furthermore, we predict sequences compatible with ubiquitin Backrub ensembles using computational protein design as implemented in Rosetta [54] and test whether these sequences are similar to the sequences of the UBQ subfamily.

Supporting our hypotheses, we find Backrub ensembles that are simultaneously consistent with native-state dynamics reflected in RDC measurements, the conformational variability observed in ubiquitin complex structures, and the conformational and sequence diversity of ubiquitin homologs. As an additional validation of our approach, we show that Backrub ensembles give similar agreement with the RDC data as ensembles generated from RDC-restrained MD simulations [4], and support previous observations of ubiquitin core flexibility [21] and binding by conformational selection [4]. Notably, we discover that a common set of Backrub sampling parameters are simultaneously able to best fit the RDC data and allow sampling of sequences most similar to those of the ubiquitin family. Our method to model Backrub ensembles and sequences consistent with these ensembles may thus be useful for providing insights into the relationship between native state dynamics and sequence diversity and for characterizing evolutionary sequence changes. These results also support the idea that Backrub ensembles will be useful for engineering new protein functions through experimental selection from computationally designed libraries [55],[56] that contain sequences accommodated by exploiting intrinsic native-state dynamics.

## Results

### Overall computational strategy

**Figure 1:**
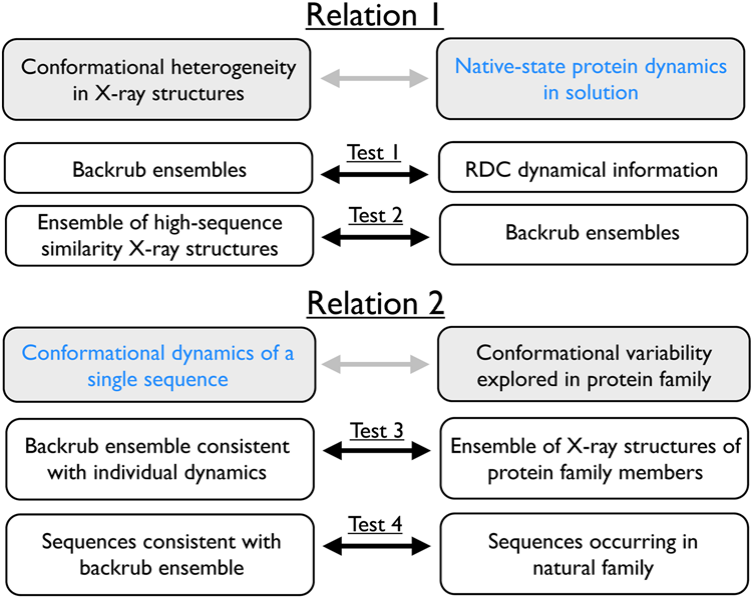
Schematic describing the two main relations evaluated in this work and the tests performed.

We set out to investigate the hypothesized relations between conformational changes reflecting observed heterogeneity in protein crystal structures, native-state protein dynamics and evolutionarily sampled conformational and sequence diversity in two steps (Figure 1).

First, to test relation 1, we generated ensemble descriptions of ubiquitin dynamics using the Rosetta scoring function and several parameterizations of the Backrub motional model (described below) without using experimental restraints. Subsequently we selected ensembles according to their agreement with RDC measurements (Test 1). This approach is significantly different from many of the methods applied earlier to find ensembles compatible with NMR restraints [4],[57],[58], which incorporated experimental data directly in the refinement process. Similar to previous work [4], we compare the resulting Backrub-generated conformational ensembles with an ensemble of 46 crystal structures of ubiquitin (Test 2).

Second, we use the insight gained from the comparison of Backrub ensembles with characteristics of solution-state dynamics to evaluate relation 2 (Figure 1). We investigate whether Backrub ensembles that sample the conformational space available on the RDC timescale have similar conformational variability to that explored by ubiquitin homologs (Test 3). Moreover, we test whether sequences consistent with Backrub ensembles fitting RDC measurements of a single ubiquitin sequence, as predicted by computational protein design using Rosetta [54], show overlap with the sequences of the natural UBQ subfamily [53] (Test 4).

### Strategy to test relation 1

**Figure 2:**
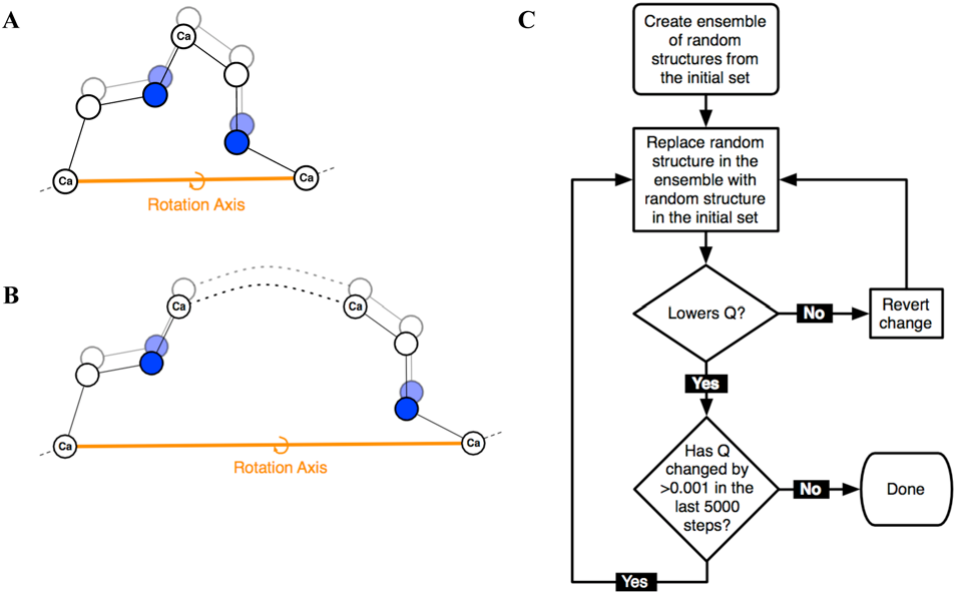
Description of the Backrub motional mechanism and ensemble selection. Backrub moves for (A) tripeptide segments and (B) segments of arbitrarily length from 2 through 12 residues. (C) Flowchart of the process used to select ensembles to match the RDC measurements.

To test relation 1, our approach first uses unrestrained conformational sampling with the Backrub motional model to generate a large set of initial conformations, starting from the ubiquitin crystal structure (Protein Data Bank (PDB) code 1UBQ). We use a Monte Carlo protocol consisting of rotamer changes and Backrub moves. Backrub moves involve selection of a random peptide segment, followed by a rigid body rotation of all atoms in that segment about an axis defined by the endpoint C-alpha atoms [31]. The peptide segment length is chosen at random to be either 2 or 3 residues (denoted in the following as “maximum segment length of 3”; Figure 2A) or between 2–12 residues (“maximum segment length of 12”; Figure 2B). 10,000 Backrub-Monte-Carlo simulations are run to generate 10,000 possible conformations in an initial set (see Methods for details). The Backrub motional mechanism thus directly accounts for correlated motions of continuous peptide segments of up to length 3 or 12. Applying these moves repeatedly in randomly chosen regions of the protein using Monte Carlo sampling allows for correlated motions of residues distant in sequence yet close in tertiary structure. Correlations between side-chain and backbone dynamics have also been observed in numerous NMR studies, such as for Ribonuclease H on the relaxation time scale [59],[60] and on the RDC time scale for ubiquitin [61] and Protein G [38].

Subsequently we select ensembles from the resulting structures based on their agreement to the RDC measurements as measured by the Q-factor (Figure 2C), defined as:
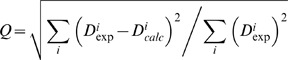



An ensemble selection approach similar to the one described above has been successfully applied to model relaxation order parameters using snapshots from MD trajectories [62]. In the following sections, “RDC-optimized” ensembles are defined as those undergoing the Q-factor optimization process described in Figure 2C and “non-RDC-optimized” ensembles are generated by choosing random ensembles of 50 structures.

To validate our approach, we compare the Backrub-generated conformational ensembles to reference methods such as snapshots from an MD simulation in explicit solvent [63] and a set of representations of the dynamics commonly used to interpret the motional information present in RDC measurements. One such representation uses the ‘model-free’ formalism, which provides five parameters describing the movement of each residue [35], [64]–[66]. Another approach is ensemble-average-restrained (EAR) molecular dynamics, in which an ensemble of molecules (the “EROS” ensemble) is optimized with respect to a molecular mechanics force field potential in combination with ensemble-average restraints on the NMR measurements, including RDCs [4]. We reason that sampling methods that result in low Q-factors more closely approximate the conformational space relevant to motions on the RDC timescale than other models that describe the experimental data less well.

### Correspondence between Backrub conformational ensembles and RDC measurements of ubiquitin dynamics (Test 1)

**Figure 3:**
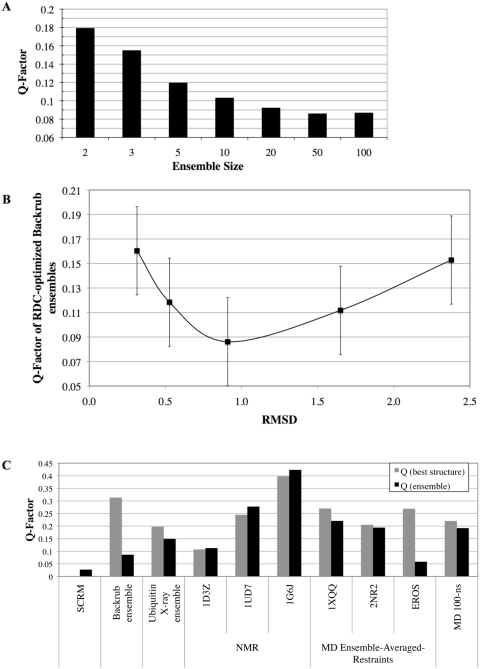
Q-factors of RDC-optimized ensembles. (A) Increasing Backrub ensemble size improves the agreement with the RDCs. Maximum segment length of 12 with kT = 1.2. (B) Q factors vs. RMSD of the RDC-optimized Backrub ensemble with the lowest Q factor at each simulation temperature for maximum segment length = 12. Error bars display Q_experimental_uncertainty_ (see Methods). (C) Q factors of the SCRM model-free description, the RDC-optimized Backrub ensemble, the ubiquitin 46-member X-ray ensemble, 3 sets of NMR structures (1G6J, 1UD7, and 1D3Z), 3 molecular dynamics simulations with ensemble-average NMR restraints (1XQQ, 2NR2, and EROS), and a 100-nanosecond MD simulation [63]. For the X-ray structures, amide hydrogen atoms were added using the Rosetta molecular modeling program with an NH bond length of 1.01 Å.

We first tested whether Q-factors of Backrub ensembles selected according to the strategy described in Figure 2C decreased as the ensemble size was increased (2, 3, 5, 10, 20, 50 and 100 structures per ensemble). This behavior would be expected if our description captures dynamical information contained in the measurements. Figure 3A shows the Q-factors of RDC-optimized ensembles of varying size generated with a Backrub maximum segment length of 12 and a simulation temperature of kT = 1.2 (see Methods). There is a clear trend that the Q-factors of RDC-optimized ensembles decrease as the ensemble size increases. This trend indicates that adding more structures allows a better representation of the RDC measurements and further suggests that these ensembles are representative of conformations that are populated on the timescale of the experiments (even though the Monte Carlo simulations are agnostic to timescale). This result is not simply explained by inclusion of more degrees of freedom and overfitting, as cross-validation analysis supports an optimal ensemble size of around 50 (Table S1). We use this ensemble size in the experiments below.

### Varying the temperature and the maximum segment length affects the agreement of RDC-optimized Backrub ensembles with RDC measurements

The RDC-optimized Backrub ensemble described above has a Q-factor of 0.086 over regions of regular secondary structure (see Methods) and was found by comparing motional models using different Backrub sampling parameters. The first Backrub parameter we varied was the maximum segment length (as described above and illustrated in Figure 2A and B, the longest peptide segment rotated about an axis defined by the segment endpoint C-alpha atoms). The Backrub conformational change observed in ultra-high resolution X-ray structures consisted of concerted 2- and 3-residue Backrub moves [29]; thus we first tested a maximum segment length of 3. In a previous study [30], we showed that ensembles of structures generated using this maximum segment length improved predictions of side-chain relaxation order parameters. To test the relevance of larger-scale changes, we also tested a maximum segment length of 12, which included moves of all intermediate segment lengths from 2–12. To measure the effect of varying the amplitude of motion, we tested a range of temperatures for the Metropolis Monte Carlo simulations from kT = 0.3 to 4.8. Each simulation was run for 10,000 steps. The resulting mean pair-wise root mean squared deviations (RMSDs) to the ubiquitin X-ray structure 1UBQ of the Backrub ensembles spanned the range of 0.2 Å to 0.5 Å for the maximum segment length of 3 simulations, and spanned the range of 0.3 Å to 3.2 Å for the maximum segment length of 12 simulations (see Methods for details).

Figures 3B shows the RDC-optimized ensembles of size 50 with lowest Q-factor for different initial Backrub starting sets of 10,000 structures with maximum segment length of 12 and different simulation temperatures. Simulation temperatures of kT = 0.3, 0.6, 1.2, 2.4 and 4.8 gave mean pair-wise RMSD values to the ubiquitin X-ray structure 1UBQ of 0.3 Å, 0.5 Å, 0.9 Å, 2.1 Å and 3.2 Å, respectively. For the maximum segment length of 12, the lowest Q factor is 0.086 at kT = 1.2 and for the maximum segment length of 3 the lowest Q factor is 0.089 at kT = 2.4 (see Table S2 for results for all parameters). To compare these two ensembles, we performed cross-validation with four RDC datasets of N-C′ couplings and four datasets of H-C′ couplings (see Methods for details). The resulting R_free_ values for these ensembles were 18.0% and 21.3%, respectively (Table S1). Thus the ensemble generated using a maximum segment length of 12 appears to be a better representation of the dynamics in the RDC measurements; we focus on this ensemble in the analyses below.

**Figure 4:**
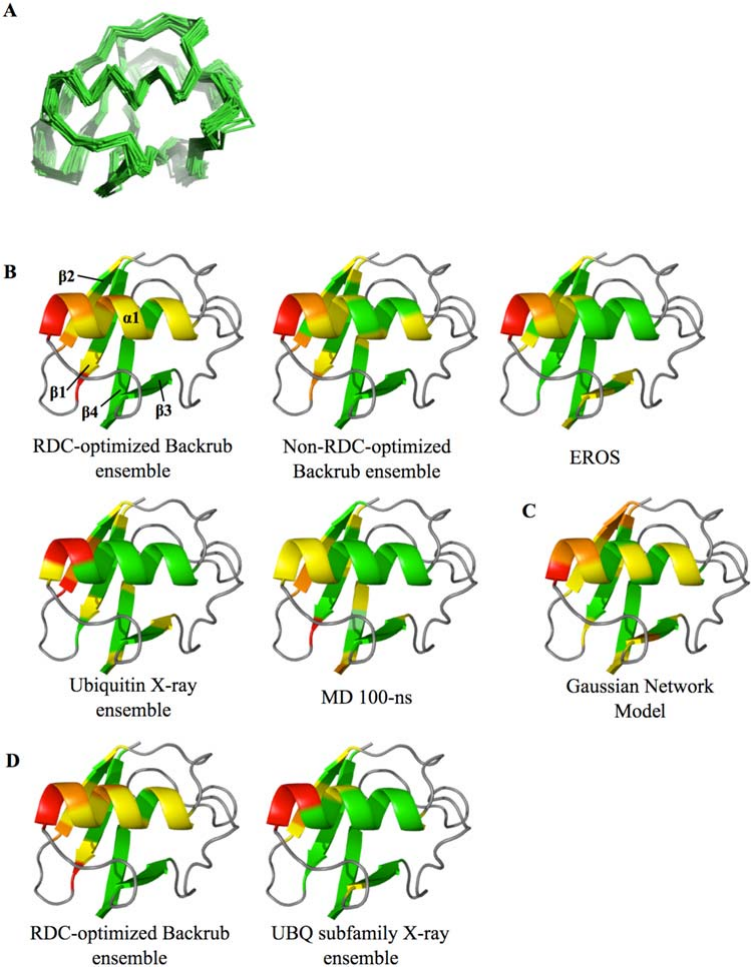
Flexibility of different ubiquitin ensembles. (A) Structures of the C-alpha backbone traces of a RDC-optimized 50-member ensemble of maximum segment length of 12 with kT = 1.2. (B and D) Mean C-alpha difference distance values of indicated ensembles mapped onto the 1UBQ X-ray structure. (C) Theoretical B-factors from a Gaussian Network Model. Color coding for B, C and D: Green: 0–25% of the max value in the non-loop regions; Yellow: 25–50% of the max; Orange: 50–75% of the max; Red: 75–100% of the max; Grey: loop regions that were not included in the fit to the RDC measurements.

The structural variability of the ensemble is illustrated in Figure 4A. The average NH order parameter in regular secondary structure elements is 0.76, the same as that computed for the model free analysis (0.77) described in Lakomek et al., but lower than that computed for the EROS ensemble (0.83) [4],[35].

### RDC-optimized Backrub ensembles match RDC measurements comparably to or better than other methods

We compared the Q-factor of the RDC-optimized Backrub ensemble to the Q-factors from various other ubiquitin ensembles (Figure 3C): the Self-Consistent RDC-based Model-free (SCRM) description (an analytical description of the RDCs with five parameters per residue that does not provide an explicit all atom structural representation of the motions) [35], an ensemble of 46 X-ray structures of ubiquitin alone and in different complexes (henceforth called the ubiquitin X-ray ensemble) as used in reference [4], three sets of NMR structures (1D3Z, 1UD7, and 1G6J), three molecular dynamics (MD) ensemble-average-restraint (EAR) ensembles (1XQQ, 2NR2, EROS PDB code 2K39) [4],[57],[58], and snapshots from a 100-nanosecond MD simulation [63]. We also examined the root mean squared error in the RDCs as a measure of quality of fit, and the results were similar (Figure S1A). The RDC-optimized Backrub ensemble has lower Q-factors than ensembles generated using other methods, except for the SCRM description [35] and the EROS ensemble, both of which were fit with the same dataset of RDC measurements as the Backrub ensembles. Not surprisingly, the SCRM Q-factor is the lowest because it is an analytical description fit to the RDCs. The EROS ensemble was created with an approach where the RDCs are incorporated into the potential function of an ensemble MD simulation and this approach gives very low Q-factors. An analysis of structural quality measures of Backrub and other conformational ensembles is given in Text S1 and Figure S2. The RDC-optimized Backrub ensembles also have similar R_free_ values from cross-validation: 18.0%, 16.1%, 20.0%, 17.8%, and 23.3%, respectively for the RDC-optimized Backrub ensemble, the EROS ensemble, the 1D3Z structures, the ubiquitin X-ray ensemble and the ensemble of MD snapshots (Table S1).

One important criterion with which the various ensembles of ubiquitin can be assessed, as mentioned above, is whether an ensemble matches the RDCs better than any single structure within it. If this is the case, dynamical information contained in the experimental measurements can be interpreted by analyzing the conformational variability in the ensemble. The RDC-optimized Backrub ensemble, the MD-EAR ensembles (1XQQ, 2NR2 and EROS PDB code: 2K39) and, the ubiquitin X-ray ensemble and the ensemble of MD structures have improved Q-factors over the best single structure (Figure 3C). The two MD-EAR ensembles that were fit to relaxation NMR measurements (1XQQ and 2NR2) have small fractional improvement in Q-factor, suggesting that the dynamic information present in the RDCs may be different from the information present on the shorter time scale relaxation measurements; this observation is supported by the different pattern of order parameters observed between these two classes of measurements [35]. The Backrub and the EROS ensembles show the largest fractional Q-factor improvement. Note that this does not contradict the fact that Backrub moves were able to improve modeling of faster time-scale picosecond-nanosecond side-chain motions [30]; the Backrub ensembles used in our previous work were not selected for agreement with the RDCs and the simulation temperature used was lower, resulting in smaller motional amplitudes.

The three sets of NMR structures (1D3Z, 1UD7, and 1G6J) do not show an improvement in the Q-factor over the best single structure. For the 1D3Z NMR structures, a subset of the RDCs were used in the refinement and, as a result, the Q-factor (Q = 0.107; calculated over all 23 datasets used in this paper) is lower than for the other NMR structures. The Q-factor of the lowest single 1D3Z NMR structure indicates that the 1D3Z NMR structure is a good representation of the average structure.

We also used the strategy described in Figure 2C to generate RDC-optimized ensembles consisting of structures from the various ubiquitin ensembles (Figure S1B). The Q-factor decreased substantially for the ubiquitin X-ray ensemble (34% lower Q-factor), the MD-EAR ensembles, (41%, 49% and 31% decrease in Q-factor for 1XQQ, 2NR2, and EROS, respectively) and the ensemble of snapshots from the 100-ns MD simulation (64% decrease). These findings are consistent with the results above that all ensemble types except the three sets of NMR structures provide insight into the RDC dynamics. The Q factors of the RDC-optimized ensembles of ubiquitin X-ray structures (Q = 0.089) and the MD snapshots (Q = 0.069) are quite similar to the Q factors of the best RDC-optimized Backrub ensemble. This latter result suggests that the 100 ns explicit water MD simulation, although short in comparison to the RDC timescale, may allow regions of ubiquitin to locally sample conformations in agreement with the RDC measurements; this is consistent with the observation from other studies that relatively short MD simulations capture a significant fraction of the motions measured by RDCs [67],[68]. Longer timescales or analyses of multiple trajectories may be needed to sample combinations of these conformations throughout the ubiquitin structure. This idea was suggested by Henzer-Wildman et al. [8] to explain the ability of adenylate kinase to sample substates in nanoseconds along the open-closed trajectory that exchanged on the order of micro- to milliseconds.

### Correspondence of conformational variability in Backrub ensembles and structural heterogeneity of ubiquitin in multiple crystal structures (Test 2)

To characterize the conformational variability of different regions of the protein in our ensembles, we calculated C-alpha difference distance matrices (see Methods and Figure S3A) [45]. These matrices show the motion of each residue with respect to all other residues. For clarity, we collapse these matrices onto a single dimension that represents the average C-alpha difference distance with respect to other residues in the protein (Figure S3B). This metric is sensitive to motions relative to those of other residues in the ensemble, as opposed to C-alpha RMSD, which is sensitive to changes relative to one conformation in the ensemble. Figures 4B and 4D show these C-alpha difference distance values mapped onto the ubiquitin structure (see Methods).

Supporting relation 1, the pattern of motion of the ubiquitin X-ray ensemble and the RDC-optimized Backrub ensemble show substantial similarities. In both these ensembles the most flexible regions are the C-terminal end of the helix and the N-terminal end of beta strand 2. This result is consistent with the suggestion of Lange et al. [4] that the native state dynamics of ubiquitin encompass the conformational flexibility found in crystal structures of ubiquitin bound to different partners, supporting a conformational selection model for binding. Moreover, the patterns of motions of the RDC-optimized Backrub ensemble are similar to the EROS and the MD ensembles despite their different amplitudes. In addition, RDC-optimized and non-RDC-optimized ensembles are similar to each other with respect to the average C-alpha difference distance matrices shown in Figure 4B. Text S1 and Figure S4 give a more detailed comparison of RDC-optimized and non-RDC-optimized conformational ensembles.

We also investigated the differences between the RDC-optimized Backrub and the ubiquitin X-ray ensemble flexibilities in light of the errors in the calculated RDC values in these regions (Figure 4B and Figure S3C). The differences in flexibility of these ensembles are mainly around the C-terminus of beta strand 1 and the alpha-helix. In the C-terminal tail of beta strand 1, residue 6 has some of the highest errors in the Backrub ensemble. Since the flexibility is low in this region in both the X-ray ensemble and the EROS ensemble, the Backrub model may overestimate the flexibility. In the helix, the relative amplitude of flexibility is also higher in the Backrub ensemble than in the X-ray ensemble; however, the pattern of flexibility is quite similar (see Figure S3C). Interestingly, the helix C-terminal residues in the X-ray ensemble show less agreement between experimental and back-calculated RDCs (Figure S3C), implying that the high flexibility in this region for the Backrub ensemble is likely to be a better representation of the RDC data. This observation agrees with the amplitude and pattern of flexibility in this region of the EROS ensemble. In addition, we observe correspondingly higher flexibility in the helix in a structural alignment of members of the ubiquitin family (Figure 4D), as discussed further below (Test 3).

As a final point of comparison, we applied a Gaussian network model (GNM) [69]. These models have been used to describe slow motions in proteins. Figure 4C shows the GNM computed B-factors mapped onto the ubiquitin structure, displaying conformational variability similar to the other methods and the X-ray ensemble, although some differences compared to the X-ray ensemble are apparent, such as along the alpha-helix and in beta strand 2.

### Structural and functional insights from ubiquitin conformational ensembles

**Figure 5:**
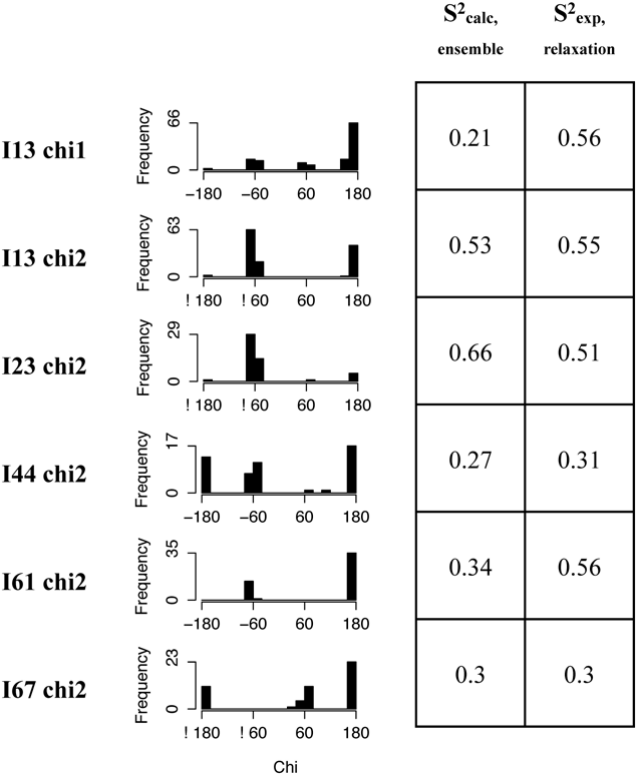
Chi angle distributions of residues in or near the core of ubiquitin. Distributions are shown for the best RDC-optimized Backrub ensemble with maximum segment length of 12 and kT = 1.2, as well as modeled and experimental relaxation order parameters corresponding to these chi angles (chi1 and chi2 correspond to the Cγ and Cδ methyl groups, respectively). The Leucine Cδ methyl group relaxation order parameters were averaged.

We showed above that our RDC-optimized Backrub ensemble (i) gives similar Q-factors to reference ensembles including an RDC-restrained MD ensemble (EROS) [4], a ubiquitin X-ray ensemble and an ensemble of snapshots from a 100-nanosecond MD trajectory [63] and (ii) has similar regions of structural variability (Figure 4B). As an additional point of comparison and validation of our approach, we asked whether the RDC-optimized Backrub ensemble also supports other structural and functional insights derived from previous ensemble descriptions of ubiquitin. Lindorff-Larsen et al. [58] as well as Richter et al. [57] used MD simulations with side chain and backbone relaxation order parameters as restraints. These ensembles displayed liquid-like flexibility of side chains buried in the protein core. The RDC-optimized Backrub ensemble also has this property, with buried or near buried residues 13, 23, 44, 61, and 67 correctly modeled as flexible with calculated order parameters close to their respective values from NMR relaxation experiments. As shown in Figure 5, Ile 13 chi2, Ile 44 chi2, and Leu 67 chi2 have modeled order parameters within 0.04 of the experimental values. Ile 13 chi 1 and Ile 61 chi2 have modeled order parameters that are substantially lower than the experimental values but these differences can be due to the short timescale of the relaxation measurements compared to the longer timescale of the RDCs fit by the RDC-optimized Backrub ensemble. (See Figure S5 for comparison to more side chains analyzed in [58].) Side chain order parameters derived from the 100 ns MD simulation discussed earlier are also shown in Figure S5 for comparison. In several cases, the side chain order parameters from the MD simulation are higher than those obtained from the relaxation experiments, possibly due to sampling limitations at the side chain level. Exceptions are the modeled order parameters for L15 chi2 and I61 chi2, which are significantly lower than the measured relaxation order parameters (this may be because the timescale of the MD simulation is longer than the rotational correlation time of the molecule).

Ubiquitin has several hotspots shown to be important in recognition of different binding partners: Ile 44, Asp 58, and His 68. These were identified as rigid in the order parameters of the EROS ensemble [4]. Residues Ile 44 and His 68 are also among the most rigid in the Backrub ensemble according to analysis by order parameters and C-alpha distance difference value (Figure S4G and S3B, respectively). Likewise the secondary structure residues observed to be most flexible by order parameters calculated from the EROS ensemble are those in the N-terminus of strand 2 which our analysis also observes to be quite flexible. We find flexible regions in the C-terminus of the alpha helix that is reflected in the C-alpha distance difference value of the EROS ensemble but not in its order parameter.

### Strategy to test relation 2

Our results above provide support for the hypothesis of a correspondence between the properties of Backrub-derived conformational ensembles, solution-state dynamics reflected in NMR measurements and a conformational ensemble of 46 experimental crystal structures of ubiquitin. To broaden this result and shed light more generally on a link between protein dynamics and evolution, we next ask whether there is also a correspondence between the dynamics of a single protein sequence and the conformational variability explored in its protein family to accommodate sequence changes during evolution (relation 2; Figure 1). In order to test this relation, we first compare the conformational variability present in the RDC-optimized Backrub ensemble with that observed in a structural alignment of 20 members of the UBQ subfamily (Test 3). Second, we extend this comparison from structural variation to sequence variation by comparing sequences modeled on Backrub ensembles to the sequences of the natural UBQ subfamily (Test 4).

### Individual and family conformational variation (Test 3)

To test the correspondence of the conformational variability of an individual protein and that of its family, we constructed an ensemble from the available structures of proteins in a multiple sequence alignment of the UBQ subfamily (see Methods for details) [53]. We performed a multiple structure alignment of this 20-member UBQ subfamily ensemble using MAMMOTH-mult [70] resulting in 66 positions that aligned in all proteins (see Methods). These aligned positions had at most 85% and an average of 21% pair-wise sequence identity. We calculated the C-alpha average distance difference matrix for these aligned positions and Figure 4D shows the average values for each residue in the matrix mapped onto the 1UBQ structure, as described for Test 2.

The resulting UBQ subfamily ensemble shows high variability in the C-terminus of the helix and in the N-terminus of beta strand 2, which is strikingly similar to the regions of high flexibility in the RDC-optimized Backrub ensemble. Thus, we find similar conformational variability in the structures of the ubiquitin homologs and in an ensemble fit to the solution state dynamics of ubiquitin. This correspondence in pattern of flexibility holds despite the different motional amplitudes of these ensembles: 2.0 Å and 0.9 Å pair-wise RMSD to the 1UBQ X-ray structure, respectively, for the UBQ subfamily ensemble and the RDC-optimized Backrub ensemble.

### Modeling of sequence space (Test 4)

We proposed in hypothesis 2 and showed above that there are similarities in the conformational variability of a single protein and that of its homologs. Here we extend this idea to ask whether the sequences compatible with a structural ensemble describing the dynamics of a single protein are similar to the sequences of the natural family members. We first tested whether there is a difference between the sequence spaces consistent with the RDC-optimized and non-RDC-optimized Backrub ensembles. We performed computational protein design with Rosetta [54] using simulated annealing of rotamer conformations and amino acid identities on each backbone in an ensemble to determine low-scoring sequences compatible with that ensemble. All positions were allowed to vary to any amino acid and 1000 low-energy sequences were generated for each ensemble. In the following, we use the term ‘sequence space’ to describe the high-dimensional space of possible sequences of a protein.

To compare the sequence space coverage of the various ensembles, we used the BLOSUM62 matrix [71] to calculate the distances between all pairs of sequences. This resulted in a distance matrix of size NxN (where N is the number of sequences compared) representing a sequence space of dimensionality N. To visualize the relative sequence space coverage of different sets of sequences we collapsed this sequence space into two dimensions using multidimensional scaling, retaining the two dimensions containing the most variation in sequence distances (see Methods).

**Figure 6:**
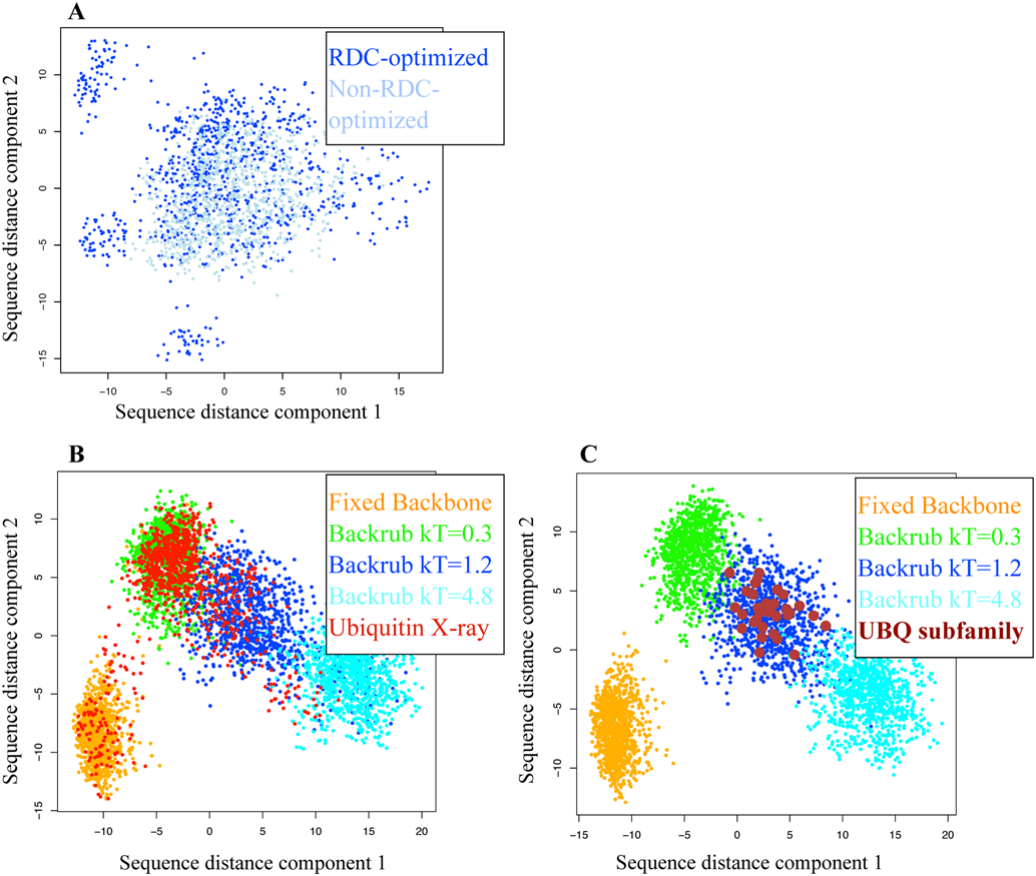
Sampling of sequence space by computational design on different ubiquitin ensembles. (A) Designed sequences on non-RDC-optimized (light blue), and RDC-optimized (dark blue) Backrub ensembles of maximum segment length of 12 with kT = 1.2. (B) and (C): Low-scoring designed sequences on the fixed backbone of the X-ray structure 1UBQ (orange); on non-RDC-optimized Backrub ensembles with maximum segment length of 12 with kT = 0.3 (green), kT = 1.2 (blue), and kT = 4.8 (cyan); and (B) low-scoring designed sequences on the ubiquitin X-ray ensemble (red), or (C) sequences from the UBQ subfamily (brown). (Note that the dimensions shown in the plots are selected to maximize the variation of the points in each plot and will differ between plots).

The sequence spaces sampled by the RDC-optimized and non-RDC-optimized Backrub ensembles with optimal Backrub parameters (maximum segment length of 12 and kT = 1.2) are very similar (Figure 6A). This is consistent with the idea that the Backrub method captures a significant portion of near-native protein motions, even without directly incorporating the RDC information into the model. In the following, we use results for non-RDC-optimized ensembles; the results are similar for RDC-optimized ensembles.

Next we compared the 2-D sequence space of designs on various non-RDC-optimized Backrub ensembles to the sequence space of designs on the ubiquitin X-ray ensemble. Different non-RDC-optimized Backrub ensembles of maximum segment length of 12 with varying amplitude (kT = 0.3, 1.2 and 4.8) sample largely separate sets of sequences (Figure 6B). Sequences move further away from the sequences sampled using the fixed backbone with increasing amplitude of motion in the ensemble. Notably, the Backrub sampling parameters used to generate ensembles which sample a range of sequences most similar to the 46-member ubiquitin X-ray ensemble are the same parameters that gave the lowest Q-factor (maximum segment length of 12 with kT = 1.2), supporting the hypothesis that the Backrub ensembles are sampling similar conformational heterogeneity to the ensemble of ubiquitin X-ray structures (Test 2). Sequences obtained from the MD ensemble are likewise most similar to the kT = 1.2 amplitude ensemble (Figure S6C and D), although spanning a somewhat larger region of sequence space.

Finally, to test whether there exists a link between the conformational heterogeneity of solution dynamical ensembles and the sequence space compatible with these ensembles (Test 4), we compared the 2-D sequence space of designs on various Backrub ensembles to the sequence space of the UBQ subfamily of the ubiquitin αβ roll subfold (Figure 6C). The subfamily sequences we used came from a high quality manually curated alignment of 36 homologues created using 3D structural analysis [53]. As shown in Figure 6C, the sequences in these naturally occurring proteins represent a subset of the sequence space of the non-RDC-optimized Backrub ensemble (maximum segment length of 12 with kT = 1.2). In contrast, the UBQ subfamily sequences barely or do not at all overlap with the sequences from design simulations using the fixed backbone, or the kT = 0.3 and kT = 4.8 ensembles. We obtain similar results when considering core residues only (Figure S6B).

**Figure 7:**
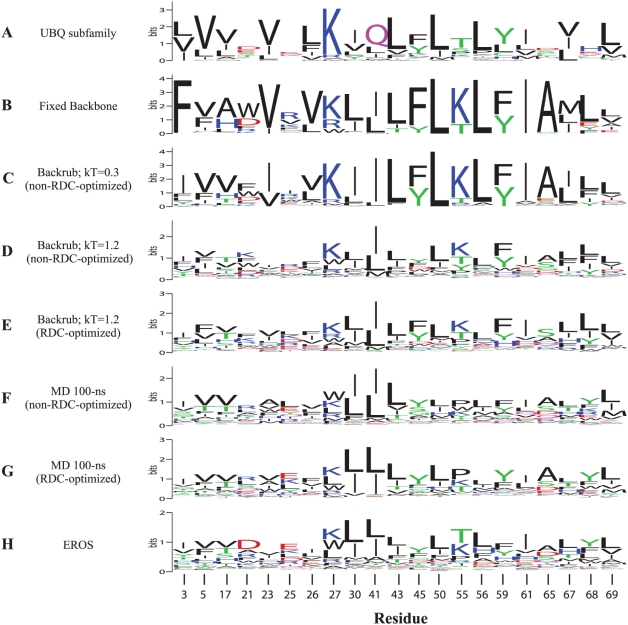
Comparison of sequence profiles of the UBQ subfamily and that of computational designs. Sequence logo plots for (A) the UBQ subfamily, and low-scoring designed sequences on (B) the 1UBQ fixed backbone, (C) the non-RDC-optimized ensemble created with maximum segment length of 12 and kT = 0.3, and (D) the non-RDC-optimized and (E) RDC-optimized ensembles with maximum segment length of 12 and kT = 1.2. Designed sequences on (F) non-RDC-optimized and (G) RDC-optimized ensembles from a molecular dynamics trajectory of 100-nanoseconds. (H) Designed sequences on the EROS ensemble. Figure created using WebLogo [82].

The sequence logo representations in Figure 7A–H for residues in buried core regions (see Methods) support the correspondence between the sequence diversity in Backrub ensembles and the natural family. The predominant amino acid in the UBQ subfamily is generally recapitulated in the non-RDC-optimized Backrub ensembles of maximum segment length 12 with kT = 0.3 and kT = 1.2 (e.g. positions 5, 27, 43, 50, 56, 61, and 69). One notable exception is that the designed sequences fail to recapitulate the frequently observed glutamine at position 41. Kiel et al. [53] use this position as the main indicator in categorizing subgroups of the UBQ subfamily because its presence correlates with the structure of a nearby loop. The side chain amide nitrogen atom of Gln 41 forms a buried hydrogen bond with the backbone of residue 36, which may be responsible for structural specificity of the loop conformation that we are not accounting for in the design simulations. Several positions, such as residues 21, 25, 45, 55, 61, 65, and 68, have high sequence entropy in the natural family. The Backrub ensemble designs recapitulate high sequence entropy for these residues. Especially for residues 45, 55, 61, and 65 the high entropy underscores one of the uses of flexible backbone design, as with a fixed backbone or low temperature Backrub ensemble only a few amino acid types predominate at those positions failing to capture the substantial natural sequence plasticity within the family. We also generated designs compatible with the trajectory of the 100-ns MD simulation, which showed similar results to the RDC-optimized Backrub ensemble overall, but with higher sequence entropy for several positions (as reflected also in Figure S6).

Taken together, our results thus indicate that the conformational sampling methods we use here to match RDC dynamics produce variability similar to the conformational heterogeneity of X-ray ensembles (both using different ubiquitin structures as well as structures from the UBQ subfamily) and may lead to significant overlap between sequences consistent with modeled ensembles and the sequence space covered by the natural family. Additionally, it appears from the similarity of sequences from RDC-optimized and non-RDC-optimized ensembles that the RDCs have led us to determine optimal Backrub sampling parameters (Figure 3B) that can be used prospectively to make modeling predictions.

## Discussion

In this work, we describe the application of the Backrub motional model to create ensembles of structures consistent with RDC measurements and to sample the conformational and sequence space of the UBQ subfamily.

The main new aspect of our work is that we link the conformational dynamics of a single sequence, as reflected by both RDC data and Backrub ensembles, to conformational diversity observed in crystal structures of ubiquitin and its family, and to evolutionary sampled sequence diversity. We achieve this by applying computational protein design to select low-energy sequences consistent with Backrub ensembles. The fact that low-Q factor Backrub ensembles sample a similar sequence space to that of the ubiquitin X-ray ensemble extends results by other groups demonstrating the correspondence of solution-state dynamics and crystallographic heterogeneity [21],[35]. In addition, we find that this designed sequence space consistent with optimal Backrub ensembles encompasses the sequence space of the UBQ subfamily, providing evidence for the idea suggested by Davis et al. [29] that the Backrub motional mechanism may facilitate amino acid changes during evolution.

We find that RDC-optimized ensembles created with only certain Backrub sampling parameters were able to reach the lowest Q-factors, indicating that the conformational space sampled by these Backrub parameters is the most similar (compared to other parameters) to the conformations giving rise to the RDC measurements. However, while we see significant improvements in Q-factors during the selection protocol, we also find substantial similarities between RDC-optimized and non-RDC-optimized Backrub ensembles in patterns of C-alpha RMSD, order parameters and designed sequence space. This somewhat surprising observation could mean that the selection procedure primarily optimizes for subtle differences in NH-vector orientations (Figure S7), while other dynamical features that are commonly characterized (such as the anisotropy of motions) are essentially indistinguishable between RDC-optimized and non-RDC-optimized Backrub ensembles. Analysis by cross-validation shows an improvement in R_free_ for RDC-optimized over non-RDC-optimized ensembles, indicating that other aspects of the peptide plane orientation are better represented in the RDC-optimized ensembles. Notably, there are defined Backrub parameters that simultaneously give the best agreement with the RDC data (after selection) and the best sequence space overlap with the natural family, irrespective of whether we apply selection or not. This could indicate that it is primarily the mechanism and amplitude of motions that are important, and that, as long as the amplitude is in the correct range defined by the appropriate sampling parameters, the Backrub motional model can sample relevant motions without requiring RDC data. Hence, the Backrub motional model may be useful (i) to predictively sample conformations similar to ensembles of bound conformations and (ii) to use with design to sample the sequence space of the natural family. Such sampling of sequences likely to be accommodated by a given protein fold may help improve engineering of new protein structures, functions and interactions. For example, coupling backbone ensemble generation and sequence design may be useful to computationally predict sequence libraries enriched in functional members [56].

There are several potential limitations of the Backrub method, as applied here. As we implement Backrub in a Monte Carlo protocol, the timescale of conformational transitions is not taken into account. Also, the method used here limits the backbone conformational space sampled to those conformations accessible with the Backrub mechanism, a restriction which can be alleviated for example with the addition of small phi/psi changes to the method or by using analytical methods for local loop closure [72], which is a superset of the Backrub move. Nevertheless, Backrub changes have an interesting similarity to the 1D-Gaussian Axial Fluctuation (GAF) analytical model, a simple motional model that has been used with success to fit RDCs [52]. A dipeptide Backrub move (a tripeptide Backrub move is shown in Figure 2A) is similar to motions described by the 1D-GAF model; thus the Backrub Monte Carlo protocol, which includes moves of longer peptide segments incorporated into a Monte Carlo scheme, can be viewed as a extension of the analytical GAF model to discrete structural ensembles.

As necessitated by the scarcity of proteins with sufficient RDC data, we limit our study here to one protein and further work is needed to extend modeling of protein native state dynamics and tolerated sequence space to more proteins. However, the usefulness of the Backrub mechanism for modeling protein motions is supported by several studies [29]–[32],[73]. Our studies on ubiquitin provide an interesting benchmark case for future analyses of the correspondence of individual and family variation.

Analysis of the generated ubiquitin Backrub ensembles allows several fundamental insights on the relationship between structure, function, sequence and dynamics. The ubiquitin core flexibility and a binding mechanism by conformational selection have been pointed out previously [4],[58]. Furthermore, our study allows characterization of differences between computationally predicted and evolved protein sequences that may lead to testable hypotheses on effects not modeled in the simulations, such as evolutionary pressures to conserve functional residues. An example is the discrepancy between the predictions and the naturally occurring glutamine residue at position 41 in ubiquitin. A likely explanation why our design simulations fail to predict this preference for glutamine is that we are not taking into account avoidance of certain non-native conformations due to evolutionary pressure enforcing structural specificity.

In conclusion, we have tested a method for sampling conformational diversity using Backrub conformational changes and shown that it can generate ensembles consistent with millisecond-timescale measurements of protein dynamics. This method is computationally more efficient than molecular dynamics-based methods, allowing it to be applied to a variety of protein modeling tasks such as sequence design. Notably, we find that the method recapitulated many of the structural properties of the RDC-optimized Backrub ensembles even when the RDC measurements were not incorporated in the sampling procedure, despite the fact that the RDCs were necessary to determine the amplitudes of motion in the Backrub ensembles. We additionally find that the sequence diversity tolerated by non-RDC-optimized Backrub ensembles is similar to that of both the ubiquitin X-ray ensemble and the UBQ subfamily X-ray ensemble. This result needs to be tested on more proteins and, if validated, should be useful in making prospective predictions to numerous applications, such as protein-protein or protein-small-molecule docking, protein interface design, and enzyme design.

## Methods

### Residual dipolar coupling measurements

The dataset of RDCs we use here consist of measurements in 23 alignment media as described in Lakomek et al. [35].

### Structure processing

For all X-ray structures, explicit hydrogen atoms were added according to standard geometry using Rosetta, and the positions of hydrogens with rotatable bonds were optimized [74]. The 46-member ubiquitin X-ray ensemble used was the same as that of [4].

### Generation of conformational ensembles

To generate protein conformational ensembles, we ran “Backrub” Monte Carlo simulations, as described in [30] and [31]. Briefly, this method randomly makes one of three types of moves: (a) a rotamer change (50% of the time), (B) a local backbone conformational changes (Backrub move) consisting of a rigid body rotation of a random peptide segment about the axis connecting the endpoint C-alpha atoms (25% of the time), or (c) a composite move with a Backrub change and one or two rotamer changes (25% of the time). After each move, the positions of the C-beta and H-alpha atoms are modified to minimize bond angle strain as described [31]. This results in bond angle changes of the main chain atoms of one to four standard deviations. The mean values and standard deviations are very similar to those computed in a set of 240 high-resolution crystal structures (better than 1.3 Å) with less than 25% sequence identity culled from the Dunbrack database [75], except for some perturbation to the N-CA-C angle (mean and standard deviations are 111.5° and 4.1° in the Backrub ensembles and 111.0° and 2.5° in the crystal structure set). See Figure S2 for details on the structural quality analysis for all structures and ensembles used in this study.

We ran a Backrub Monte Carlo simulation at kT = 0.1 from the starting PDB conformation (using 1UBQ, which has the highest resolution (1.8 Å) of the unbound ubiquitin structures; similar results were obtained for maximum segment length of 3 with PDB entries 1UBI and 1CMX and worse Q factors were obtained for PDB entries 1FXT, 1AAR, 1F9J, and 1TBE) for 10,000 steps with a maximum segment length of 3 or 12, matching the segment length used later. The lowest energy structure from this simulation is used as the starting conformation for 10,000 randomly seeded Backrub simulations at one of 5 different temperatures (kT = 0.3, 0.6, 1.2, 2.4, or 4.8) run for an additional 10,000 steps. The last structure from each of these simulations is used to form the starting set of 10,000 structures.

From this initial set of 10,000 structures, ensembles are selected to match the RDCs by minimizing the Q-factor of the ensemble. First, structures are randomly chosen to create a starting ensemble of a given size (2, 3, 5, 10, 20, 50 or 100 structures), and the Q-factor of the ensemble is calculated (see below). Next, a random structure in this ensemble is chosen and replaced with a randomly chosen structure from the initial ensemble of 10,000 structures; then the new Q-factor of the ensemble is calculated. If the new Q-factor is lower than before the replacement, the change is kept, otherwise it is reverted. These structure replacements are repeated until the Q-factor changes by less than 0.001 in 5000 steps. By repeating this method 1000 times, 1000 RDC-optimized Backrub ensembles are created. There are a very large number of possible subsets of a given size. For example, there are 4*10^^^61 different sub-ensembles of size 20 from the initial ensemble of size 10,000, too many to be evaluated. The approach described here does not guarantee that the ensemble with the lowest Q-factor will be found, but it starts from many random starting points to broadly sample the space of possible sub-ensembles and the selection process converges to a low Q-factor solution within 10,000 Backrub-generated structures for all Backrub Monte Carlo temperatures (except kT = 4.8; see Figure S8).

### Calculating RDCs from a structure or structural ensemble

RDCs are calculated from a single structure and an ensemble of structures as described in [76]. Briefly, we first find the alignment tensor from a structure (or set of structures) and the experimental couplings. This is done using the equation T = A^−1^ D_exp_, where T is the alignment tensor, A^−1^ is the Moore-Penrose inverted matrix of projection angles for the amide bonds (or averaged projection angles for a set of structures), and D_exp_ is the vector of experimental couplings. The predicted couplings are then calculated with the equation D_calc_ = AT where A is the same matrix of projection angles from above and D_calc_ is the vector of calculated couplings.

Q-factors were calculated for all RDC measurements with the equation:
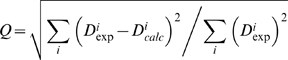



Errors between experimental and predicted RDCs were calculated with:
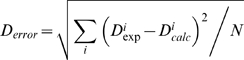



Loop residues (i.e. those with DSSP [77] secondary structure type not H, E, G or I) are excluded from the analysis in both tensor determination and back-computation of RDCs and Q-values. The non-loop residues used in all analyses in this paper are ubiquitin residues 2–7, 12–16, 23–34, 38–45, 48–49, 57–59, and 66–71.

### Sources of error

There are several sources of error in our analysis to consider when assessing the significance of the results. First, there is error in the RDC measurements due to experimental uncertainty. The uncertainty in these values is estimated to be 0.3 Hz [35]. To calculate the resulting uncertainty in the Q-factor, we added Gaussian-distributed noise of mean amplitude 0.3 Hz to the RDC measurements (see section below) in 1000 Monte Carlo trials. This resulted in a value of Q_experimental_error_ = 0.036.

A second source of error results from not finding the ensemble with the lowest possible Q-factor from a given initial structure set. We estimated this error by repeating the selection procedure many times and evaluating the variance in the resulting Q-factors. We take explicit steps to minimize this error by enforcing two convergence criteria on the optimization: 1) ensemble selection is not finished until 5000 steps have passed without a change in Q of more than 0.001, and 2) enough RDC-optimized ensembles are generated from random starting structures such that the difference in the Q-factors of the best and 10th best RDC-optimized ensemble is not more than 0.005. Thus, this Q_optimization_error_ is on the order of 0.005.

A third important source of error is due to insufficient sampling of conformational space with the Backrub Monte Carlo protocol and the 10,000 structures that we use to select ensembles from. We estimated this Q_sampling_error_ by running the structure generation protocol at each temperature 10 times, thus creating 10 sets of 10,000 Backrub-generated structures at each temperature. The standard deviations of the minimum Q-factors over these 10 sets of 10,000 structures are 0.0151, 0.0104, 0.0025, 0.0039, and 0.0049 for kT = 0.3, 0.6, 1.2, 2.4 and 4.8, respectively for a maximum segment length of 12. The standard errors of the mean of these values are 0.0048, 0.0033, 0.0008, 0.0012, and 0.0015, respectively.

### Calculation of the experimental uncertainty in the Q-factor (Q_experimental_uncertainty_)

Gaussian-distributed noise was added to the experimental RDCs with 1000 Monte-Carlo samples. The RDC uncertainty of each measurement was 0.3 Hz [35], which was used as the standard deviation of the Gaussian noise function. The resulting Q_experimental_uncertainty_ is 0.036 with a standard deviation of 0.00102 over the 1000 samples.

### Order parameter calculation

Order parameters were calculated with the equation
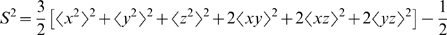
where x, y and z are the coordinates of the normalized unit vectors representing the amide bond vector orientations [78]. For the Backrub ensemble, these values were then scaled by 1/1.12 = 0.89 to account for librational effects that cannot be sampled by the Backrub method when considering only one type of RDCs [79].

### Molecular dynamics trajectory

We used the 100-nanosecond AMBER trajectory of ubiquitin in TIP4Pw/e water from Wong and Case [63]. The protein was allowed to equilibrate over the first 4.32 nanoseconds, and snapshots were taken from the following 100 nanoseconds at 10-picosecond intervals. This resulted in 10,000 structures, which were used to calculate an overall Q-factor for the trajectory. In addition, we applied the selection scheme in Figure 2C on these 10,000 snapshot structures to select ensembles with optimized Q-factors.

### Measurement of sequence space sampling

To estimate the sequence space compatible with different structures and ensembles, we used Rosetta computational protein design to generate 1000 low-energy sequences for each single structure or 20 sequences per ensemble member for ensembles of size 50. To find a low-scoring sequence, each design simulation consists of 20 rounds of Monte Carlo simulated annealing with the number of steps in each round equal to the number of rotamers created for the simulation. The backbone of each structure or ensemble member is kept fixed during the design simulations and all positions were allowed to vary to any of the 20 naturally occurring amino acids, adding extra conformers at one standard deviation around the mean rotamer for chi 1 and 2 dihedral angles. The scoring function used was the Rosetta all-atom scoring function [54], which is dominated by a Lennard-Jones potential, a geometry-dependent hydrogen-bonding potential [74] and an implicit solvation potential [80].

Distances between sequences were calculated as in [50]. Briefly, these distances were calculated as the sum of the substitution costs (using the BLOSUM62 matrix after normalizing it to range from 0 to 1) [71] for the positions that aligned in all sequences: residues 1–9, 12–24, 26–35, 40–53, 55–63, 65–71. After calculating the distances between all pairs of sequences within each ensemble and between pairs of ensembles, we used metric multidimensional scaling in R [81] to reduce the dimensionality of the space to the two dimensions spanning the most sequence distance.

The procedure was repeated with the sequences of core residues only, where core residues were defined by counting the number of neighbor residues with C-beta atoms within 10 Å of the C-beta atom of the residue of interest (or C-alpha atoms for glycine). The cutoff value used (greater than or equal to 18) was chosen so that approximately one third of the residues fell into the core category (excluding the flexible C-terminus), resulting in 21 buried positions: residues 3, 5, 17, 21, 23, 25, 26, 27, 30, 41, 43, 45, 50, 55, 56, 59, 61, 65, 67, 68, and 69.

### C-alpha difference distance matrices

First, for each structure, we calculated the matrix of distances between all C-alpha atoms. Then, for each pair of structures, we calculated the distance difference matrix as the absolute value of the difference of the distance matrices of the structures. These distance difference matrices were averaged to give the C-alpha difference distance matrix of the ensemble [45].

### Gaussian Network Model

Theoretical B-factors were calculated by applying the online Gaussian Network Model (oGNM) tool at http://ignm.ccbb.pitt.edu/GNM_Online_Calculation.htm [69] to PDB structure 1UBQ using 1 node per residue and a cutoff of 10 Å for amino acid pairs.

### UBQ subfamily structural alignment

To create a structural ensemble for the UBQ subfamily we took the highest resolution X-ray structure for each protein listed in Table 1 of Kiel et al. [53] (or the first structure of an NMR ensemble if no X-ray structure was available). We removed structures that had 100% sequence identity to other structures in the ensemble. We performed a multiple structural alignment using MAMMOTH-mult [70] and removed PDB id 1WIA because it was missing residues that aligned with part of the helix in the native ubiquitin sequence; all other structures had residues that aligned with all the residues in the secondary structure regions of ubiquitin. The resulting ensemble consisted of 20 structures: 1XD3 chain B, 1BT0 chain A, 1EUV chain B, 1IYF, 1J8C, 1LM8 chain B, 1M94, 1NDD chain A, 1OQY, 1P1A, 1TGZ chain B, 1V5O, 1V5T, 1V86, 1WE6, 1WE7, 1WGD, 1WGG, 1WH3, and 1WM3 chain A. To create the C-alpha distance difference matrix we used the 66 positions that aligned in all 20 structures, which were (using 1UBQ numbering): 1–7, 9–16, 18–34, 36–46, 48–55, 57–64, 66–72.

### Cross-Validation

We performed cross-validation by using the alignment tensor calculated from the NH RDC datasets to calculate RDCs for four datasets of NC′ RDC couplings and four datasets of HC′ couplings. These “free” data were not included in the selection process and are reported as R_free_ factors, as calculated by Lange et al. [4].
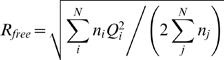
for the N different types of experiments with n_i_ measurements each and Q-factor Q_i_. For RDC-optimized Backrub ensembles, the R_free_ values are averaged over the five lowest-Q factor ensembles.

## Supporting Information

Text S1 Supplementary results & supplementary methods.
(0.06 MB PDF)

Figure S1 RDC_error_ and Q-factors of different ensembles. (A) Error in the calculated RDCs. (B) Same data as Figure 3C in the main manuscript with the addition of bars showing the minimum Q factors of RDC-optimized ensembles of size 50 (allowing multiple instances of the same structure) from the given source using the optimization approach outlined in Figure 2C of the main manuscript.
(0.58 MB EPS)

Figure S2 Stereochemistry of Backrub and other ensembles.
(0.27 MB EPS)

Figure S3 C-alpha difference distance matrices. (A) C-alpha difference distance matrices of various ensembles. (B) Mean C-alpha difference distance values for various ensembles. Red dashed lines: anchor residues 44, 58 and 68. (C) Normalized C-alpha difference distance values and RDC errors over sequence for the ubiquitin X-ray ensemble and the RDC-optimized Backrub ensemble. (The C-alpha difference distance values were normalized to the maximum and minimum values in the secondary structure regions longer than 3 residues.)
(4.29 MB TIF)

Figure S4 C-alpha RMSD and amide order parameter traces of Backrub ensembles. C-alpha RMSD traces of the best five RDC-optimized (grey) and one non-RDC-optimized (black) Backrub ensembles for maximum segment length of 3 with (A) kT = 0.3, (B) kT = 2.4, and (C) kT = 4.8 and maximum segment length of 12 with (D) kT = 0.3, (E) kT = 1.2, and (F) kT = 4.8. (G) Amide order parameters for the RDC-optimized and non-RDC-optimized Backrub ensembles, the SCRM description, the relaxation experiments, and the EROS ensemble.
(1.42 MB EPS)

Figure S5 Chi angle distributions of various residues. For the DER ensemble (1XQQ), RDC-optimized and non-RDC-optimized Backrub ensembles with maximum segment length of 12 with kT = 1.2. Also included are the order parameters for the RDC-optimized ensemble, the MD trajectory and the experimental relaxation measurements, where available.
(0.54 MB EPS)

Figure S6 Sampling of sequence space by computational design for both core only and aligned residues. Low-scoring designed sequences on the fixed backbone of the X-ray structure 1UBQ (orange); on non-RDC-optimized Backrub ensembles with maximum segment length of 12 with kT = 0.3 (green), kT = 1.2 (blue), and kT = 4.8 (cyan); and sequences from the UBQ family (brown) for (A) aligned and (B) only core residues; or low-scoring designed sequences on the 100 ns MD ensemble (red) for (C) aligned and (D) only core residues.
(1.35 MB EPS)

Figure S7 Amide vector orientations. Angle difference between the average amide vector orientation of the 1D3Z NMR ensemble and the average amide vector orientations in RDC-optimized and non-RDC-optimized Backrub ensembles (A) maximum segment length of 12 with kT = 1.2 and (B) maximum segment length of 3 with kT = 2,4. The angle difference of the average amide vector orientation of the 1D3Z ensemble is also compared to the orientation of amide vectors in two X-ray structures (with hydrogens added using Rosetta). (C) The difference in the angle differences from (A) and (B) for non-RDC-optimized minus RDC-optimized ensembles in secondary structure regions. (D) Angle differences of the two (E) RDC-optimized and (F) non-RDC-optimized Backrub ensembles plotted relative to each for residues in secondary structure regions.
(1.46 MB EPS)

Figure S8 Convergence of Q factors in the optimization protocol.
(0.17 MB EPS)

Table S1 Cross-validation analysis.
(0.04 MB DOC)

Table S2 Q-factors of RDC-optimized ensembles at various simulation temperatures and maximum segment lengths.
(0.03 MB DOC)
