# Supplementary material for: A Correspondence Between Solution-State Dynamics of an Individual Protein and the Sequence and Conformational Diversity of its Family
Source: PLoS Comput Biol. 2009 May 29;5(5):e1000393. doi: 10.1371/journal.pcbi.1000393 (PMC2682763; doi:10.1371/journal.pcbi.1000393)
Supplement: Text S1 — Supplementary results & supplementary methods. (0.06 MB PDF) [file pcbi.1000393.s001.pdf]

Text S1

SUPPLEMENTARY RESULTS

Structure quality of Backrub ensembles

In order to evaluate structural quality parameters of the different conformational ensembles, we used MolProbity [1]. The structure quality metrics of the RDC-optimized Backrub ensemble are generally within the range of values of the X-ray and NMR structures and other ubiquitin ensembles (Figure S2). For example, less than 5% of dihedrals are outside of the favored Ramachandran region in the Backrub ensembles, compared to 0-14% for different X-ray structures, 0-8% for NMR structures, 6-8% for the MD ensemble-averaged-restrained structures, and 4% for the MD structures. Some distortions are observed when the Backrub simulation temperature is increased to kT=4.8 for maximum segment length of 3 and kT=2.4 for maximum segment length of 12, where the number of steric clashes, the occurrence of residues in the non-favored Ramachandran regions and the fractional volume increase over the crystal structure have higher values than typical for the other ensemble types. Nevertheless, the Backrub ensembles that fit the RDC data best (Figure 3 in the main manuscript) appear to have reasonable geometries.

Differences between RDC-optimized and non-RDC-optimized Backrub ensembles

The Q-factors of RDC-optimized ensembles are substantially lower than the Q-factors of the non-RDC-optimized ensembles (ranging from Q=0.081 to 0.15 and Q=0.27 to 0.35, respectively, for different Backrub ensembles with maximum segment length of 3; and from Q=0.086 to 0.15 and Q=0.25 to 0.53, respectively, for maximum segment length of 12). Thus, we investigated a range of structural and dynamical parameters to characterize differences between RDC-optimized and non-RDC-optimized ensembles. Figure S4A-F show the C-alpha mean pair-wise RMSD of RDC-optimized and non-RDC-optimized Backrub ensembles of different maximum segment length and amplitude of motion. Interestingly, for the Backrub sampling parameters that yield the lowest Q-factors after selection (kT=2.4 with maximum segment length of 3 and kT=1.2 with maximum segment length of 12; see Figure 3B in the main manuscript), the patterns of

C-alpha variation of the RDC-optimized and non-RDC-optimized ensembles are comparable even while the amplitude is different (Figure S4A-F). In addition, the pattern of motion using a C-alpha difference distance analysis (see Methods and main manuscript Figure 4B) is also similar between the RDC-optimized and the non-RDC-optimized ensembles.

The order parameters between non-RDC-optimized and RDC-optimized ensembles are generally similar as well (Figure S4G), apart from some smaller differences in the helix for maximum segment length of 3 with kT=1.2 and differences in beta strand 4 of ensembles from both maximum segment lengths. Nevertheless, the correspondence in C-alpha RMSD and overall order parameter patterns between RDC-optimized and non-RDC-optimized ensembles suggest that more subtle differences – i.e. the character of the motion and not its amplitude – account for the significant differences in Q-factors.

Thus we looked in detail at the effect of ensemble selection on the properties of the amide bond vectors. Figures S7A and S7B show the difference in angle of the average amide bond vector orientations of Backrub ensembles relative to the average amide bond vector orientations in the 1D3Z ensemble (which was also fit to a subset of the RDC data). Looking at the change in this angular difference from non-RDC-optimized to RDC-optimized ensembles (Figure S7C) the orientations change in the RDC-optimized ensembles and move closer to the orientations in the 1D3Z ensemble. These angular differences are also more similar between the two RDC-optimized ensembles with different maximum segment length (R^2=0.68; Figure S7D) than between the two non-RDC-optimized ensembles (R^2=0.42; Figure S7E).

SUPPLEMENTARY METHODS

Structure quality analysis

MolProbity [1] was used for analysis of the following structural quality metrics: the number of clashes greater than 0.4Å, orientation of C-beta atoms, rotamer conformations with less that 1% frequency of occurrence in the PDB, phi/psi dihedral angles in the "core", "allowed" and "outlier" regions (defined as the top 98% of residues, the top 99.5% of residues, and the remaining residues, respectively) [2], and bond lengths and angles of heavy main-chain atoms that are more than 4 standard deviations from their expected values (Vincent Chen, personal communication). The calc-volume tool [3,4] was used to calculate the packing volume of the various structures. The values shown for ensembles are the means over the structures in the ensemble.

Analysis of amide vector orientation

To plot the amide vector orientations in the Backrub ensembles, we used the NH orientations from the 1D3Z NMR structures as a reference. For each residue we calculated the average orientation of the NH vectors in the 1D3Z structures and the average orientation of the vectors in the Backrub ensemble. We then calculated the angle between these average vectors.

REFERENCES

1. Davis IW, Leaver-Fay A, Chen VB, Block JN, Kapral GJ, et al. (2007) MolProbity: all-atom contacts and structure validation for proteins and nucleic acids. Nucleic Acids Res 35: W375-383.
2. Lovell SC, Davis IW, Arendall WB, 3rd, de Bakker PI, Word JM, et al. (2003) Structure validation by Calpha geometry: phi,psi and Cbeta deviation. Proteins 50: 437-450.
3. Harpaz Y, Gerstein M, Chothia C (1994) Volume changes on protein folding. Structure 2: 641-649.
4. Gerstein M, Tsai J, Levitt M (1995) The volume of atoms on the protein surface: calculated from simulation, using Voronoi polyhedra. J Mol Biol 249: 955-966.
