# Supplementary figures and images for: A Correspondence Between Solution-State Dynamics of an Individual Protein and the Sequence and Conformational Diversity of its Family
Source: PLoS Comput Biol. 2009 May 29;5(5):e1000393. doi: 10.1371/journal.pcbi.1000393 (PMC2682763; doi:10.1371/journal.pcbi.1000393)

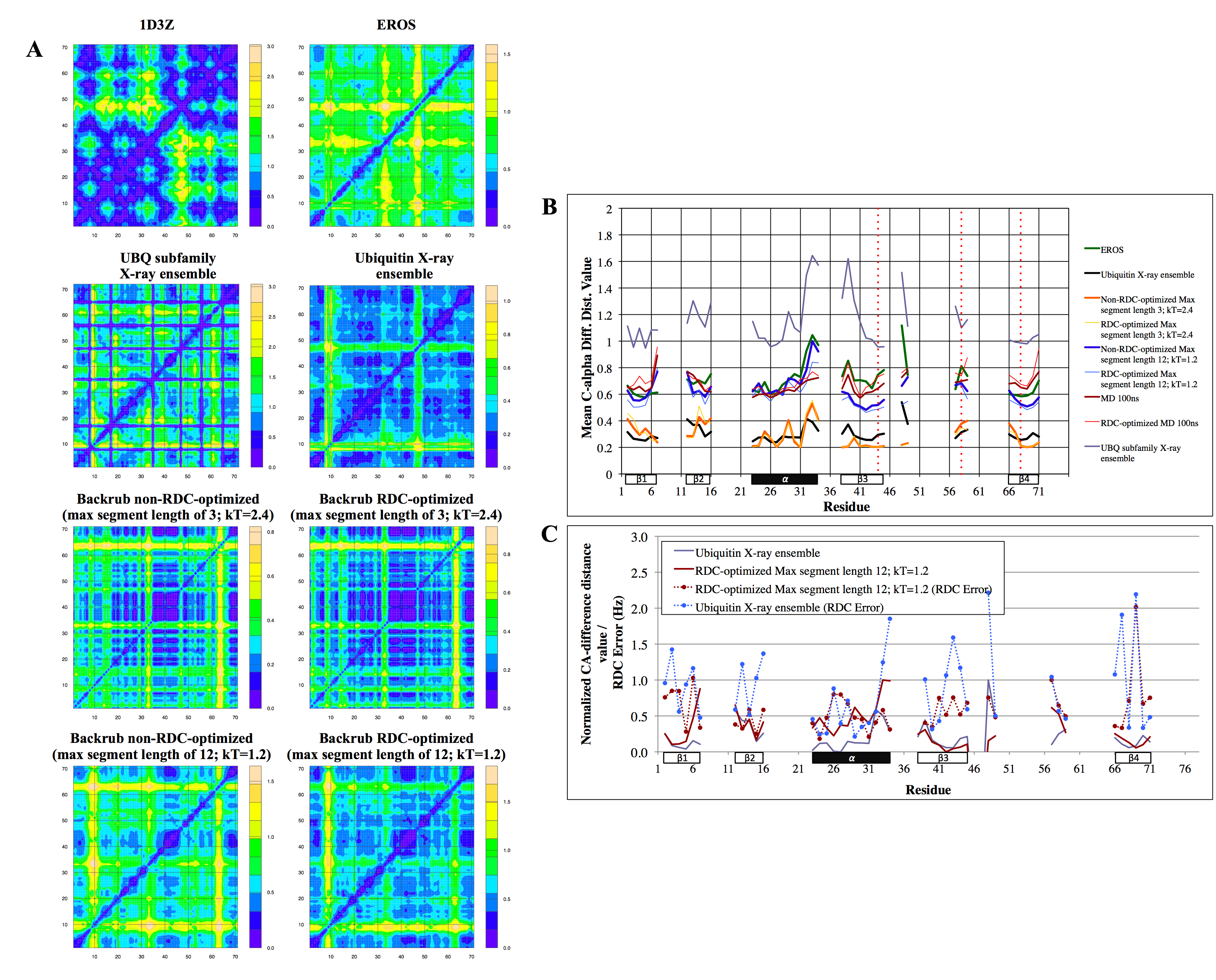

Supplement: Figure S3 — C-alpha difference distance matrices. (A) C-alpha difference distance matrices of various ensembles. (B) Mean C-alpha difference distance values for various ensembles. Red dashed lines: anchor residues 44, 58 and 68. (C) Normalized C-alpha difference distance values and RDC errors over sequence for the ubiquitin X-ray ensemble and the RDC-optimized Backrub ensemble. (The C-alpha difference distance values were normalized to the maximum and minimum values in the secondary structure regions longer than 3 residues.) (4.29 MB TIF) [file pcbi.1000393.s004.tif]
