# Supplementary material for: A Correspondence Between Solution-State Dynamics of an Individual Protein and the Sequence and Conformational Diversity of its Family
Source: PLoS Comput Biol. 2009 May 29;5(5):e1000393. doi: 10.1371/journal.pcbi.1000393 (PMC2682763; doi:10.1371/journal.pcbi.1000393)
Supplement: Table S1 — Cross-validation analysis. (0.04 MB DOC) [file pcbi.1000393.s010.doc]

**Supp Table 1**. **Cross-validation analysis.**

| **Ensemble** | **R_free_** |
| --- | --- |
| 1XQQ | 23.1% |
| 2NR2 | 19.5% |
| 2K39 | 16.1% |
| 1D3Z | 20.0% |
| 1G6J | 38.1% |
| 1UD7 | 28.3% |
| Ubiquitin X-ray ensemble | 17.8% |
| Non-RDC-optimized Backrub;  maximum segment length 12  with kT=1.2 | 20.5% |
| Non-RDC-optimized Backrub;  maximum segment length 3  with kT=2.4 | 26.3% |
| MD 100ns | 23.3% |

| **Ensemble size** | **2** | **3** | **5** | **10** | **20** | **50** | **100** |
| --- | --- | --- | --- | --- | --- | --- | --- |
| Max segment length 3 with kT=2.4 | 23.5% | 25.8% | 21.8% | 21.9% | 21.1% | 21.3% | n.d. |
| Max segment length 12 with kT=1.2 | 24.1% | 22.0% | 20.7% | 18.4% | 18.6% | 18.0% | 18.1% |

| **kT** | **0.3** | **0.6** | **1.2** | **2.4** | **4.8** |
| --- | --- | --- | --- | --- | --- |
| Max segment length 3 with ensemble size 50 | 18.8% | 18.6% | 19.3% | 21.3% | 24.8% |
| Max segment length 12 with ensemble size 50 | 18.8% | 17.8% | 18.0% | 21.6% | 27.5% |

n.d.: not determined
