# Supplementary material for: A Correspondence Between Solution-State Dynamics of an Individual Protein and the Sequence and Conformational Diversity of its Family
Source: PLoS Comput Biol. 2009 May 29;5(5):e1000393. doi: 10.1371/journal.pcbi.1000393 (PMC2682763; doi:10.1371/journal.pcbi.1000393)
Supplement: Table S2 — Q-factors of RDC-optimized ensembles at various simulation temperatures and maximum segment lengths. (0.03 MB DOC) [file pcbi.1000393.s011.doc]

**Supp Table 2.** **Q-factors of RDC-optimized ensembles at various simulation temperatures and maximum segment lengths.**

| **kT** | **0.3** | **0.6** | **1.2** | **2.4** | **4.8** |
| --- | --- | --- | --- | --- | --- |
| Max segment length=3 | 0.153 | 0.108 | 0.093 | 0.089 | 0.098 |
| Max segment length=12 | 0.160 | 0.118 | 0.086 | 0.112 | 0.153 |
